# Prognostic score to predict mortality during TB treatment in TB/HIV co-infected patients

**DOI:** 10.1371/journal.pone.0196022

**Published:** 2018-04-16

**Authors:** Duc T. Nguyen, Helen E. Jenkins, Edward A. Graviss

**Affiliations:** 1 Houston Methodist Hospital Institute, Houston, Texas, United States of America; 2 Department of Biostatistics, Boston University School of Public Health, Boston, Massachusetts, United States of America; Central University of Tamil Nadu, INDIA

## Abstract

**Background:**

Estimating mortality risk during TB treatment in HIV co-infected patients is challenging for health professionals, especially in a low TB prevalence population, due to the lack of a standardized prognostic system. The current study aimed to develop and validate a simple mortality prognostic scoring system for TB/HIV co-infected patients.

**Methods:**

Using data from the CDC’s Tuberculosis Genotyping Information Management System of TB patients in Texas reported from 01/2010 through 12/2016, age ≥15 years, HIV(+), and outcome being “completed” or “died”, we developed and internally validated a mortality prognostic score using multiple logistic regression. Model discrimination was determined by the area under the receiver operating characteristic (ROC) curve (AUC). The model’s good calibration was determined by a non-significant Hosmer-Lemeshow’s goodness of fit test.

**Results:**

Among the 450 patients included in the analysis, 57 (12.7%) died during TB treatment. The final prognostic score used six characteristics (age, residence in long-term care facility, meningeal TB, chest x-ray, culture positive, and culture not converted/unknown), which are routinely collected by TB programs. Prognostic scores were categorized into three groups that predicted mortality: low-risk (<20 points), medium-risk (20–25 points) and high-risk (>25 points). The model had good discrimination and calibration (AUC = 0.82; 0.80 in bootstrap validation), and a non-significant Hosmer-Lemeshow test p = 0.71.

**Conclusion:**

Our simple validated mortality prognostic scoring system can be a practical tool for health professionals in identifying TB/HIV co-infected patients with high mortality risk.

## Introduction

Tuberculosis (TB) is a leading cause of morbidity and mortality in HIV-infected individuals. In 2016, there were an estimated one million new TB cases amongst people who were HIV-positive worldwide with 374,000 deaths [1]. As one of the four states (California, Texas, New York, and Florida) that accounted for 50.6% of the national total cases in the United States (U.S.) [2], Texas had 9,007 new TB cases and 30,979 HIV-positive individuals reported between 2010 and 2016 [3–5]. In 2015, Texas had a TB incidence of 4.9 per 100,000 population, 5.1% of these new TB patients were HIV positive [4]. In Texas, TB disease has been identified as a communicable disease having the highest standardized mortality ratio (SMR) relative to the national reference with 679 deaths between 2001 and 2010 [6]. Studies in other settings have identified several prognostic factors associated with mortality in TB/HIV patients such as age≥45, smear(+) pulmonary TB, antiretroviral therapy (ART), having initial TB regimen with rifamycin, isoniazid and pyrazinamide, drug susceptibility testing (DST), and CD cell count [7,8]. Podlekareva et al. recently presented a health care index (HCI) score with components selected from commonly-used interventions and suggested its association with the outcome in HIV-positive patients [8]. However, the HCI components were subjectively selected and not the result of objective multivariate regression modeling or statistical analyses. Although some TB mortality risk scores have been developed, they either do not include HIV-infected patients, have small sample size, or were developed based on single hospital to predict in-hospital mortality, or include variables that are not routinely available at community TB programs [9–11]. In light of this, making an accurate prognosis for mortality risk during TB treatment in HIV co-infected patients is challenging for health professionals, especially in a low TB prevalence population, due to the lack of a standardized prognostic system. The current study aimed to develop and validate a simple prognostic scoring system using population-based surveillance data, which are routinely collected by most TB programs and could predict patient mortality risk during TB treatment. The proposed mortality risk score could be a practical tool for TB clinicians and other health professionals in managing TB disease in patients with TB/HIV co-infection.

## Methods

### Study population

The study used retrospective de-identified surveillance data of all confirmed TB patients from the state of Texas (U.S.) reported to the National TB Surveillance System (NTSS). The dataset was downloaded from the Centers for Disease Control and Prevention (CDC) supported TB Genotyping Information Management System (TBGIMS) website. The inclusion criteria were defined as: (1) confirmed TB cases in the state of Texas from 01/2010 through 12/2016 (based on the date of “caseness” when the case was verified by the Texas Department of State Health Services included in the state’s official case count) [12]; (2) age≥15 years old; (3) positive HIV status; and (4) had documented TB treatment outcome in the dataset as either treatment completed (“completed”) or dead (“died”). As the dataset has only one pediatric TB/HIV co-infected patient, this patient was not included in this study. Given the main purpose of our study is to predict the mortality during TB treatment in HIV-infected patients against the treatment completion, patients who had an outcome coding other than “completed” or “died” (such as “adverse”, “lost”, “moved”, “other”, “refused”, or “unknown”), i.e. vital status could not be verified, and had a negative or unknown HIV status were also excluded from the analyses. A confirmed TB case in the dataset is defined as either a laboratory confirmed case or a clinical confirmed case, which was identified and verified by the local and state TB program staff using the CDC’s TB case definition [12]. A patient with a *Mycobacterium tuberculosis* (*Mtb*) culture conversion was defined as a patient who had an initial positive sputum culture that converted to a documented negative culture without converting back to positive culture during the entire treatment course. Unknown conversion status was defined as a patient who had an initial *Mtb* positive sputum culture and completed TB treatment but the results of all follow-up cultures are not available. Abnormalities consistent with TB disease on chest radiograph (TB-CXR) were recorded in the dataset as a binary variable (normal versus abnormal) [13].

### Ethics statement

As this was a retrospective study using de-identified data, ethical approval was not required.

### Statistical analysis

Demographic and clinical data were reported as frequencies and proportions. Differences in demographic and clinical characteristics between the excluded and included patient population pools were determined using the Chi-square or Fisher’s exact tests, as appropriate. Missing data were assessed for missing completely at random (MCAR) and covariate-dependent missingness (CDM) using the Little’s chi-squared test [14]. Univariate and multiple logistic regression models were used to determine the contribution of potential prognostic variables to the patient outcome. Variables for multiple logistic regression models were selected using the Bayesian model averaging (BMA) method [15,16]. Briefly, Stata’s BMA program was run to evaluate possible model sets from all variables having a p-value of <0.2 in the univariate analysis or variables deemed as clinically important. The BMA program suggested good models which included the variables with a high probability of being a risk factor. The Likelihood Ratio test was used to further reduce the model subsets. The best model was selected based on the small Bayesian information criterion (BIC). Significant risk factors were assigned weighted-points that were proportional to their β regression coefficient values. A prognostic score was calculated for each individual patient in the cohort. Patients were categorized in deciles of risk score and then collapsed into three groups which were significantly distinct in predictive risk for mortality (low, medium and high risk). Model discrimination was determined by the area under the receiver operating characteristic (ROC) curve (AUC). The model’s good calibration was determined by a non-significant Hosmer-Lemeshow’s goodness of fit test. Model validation was performed using the bootstrap resampling method with 2000 replications. All the analyses were performed using Stata version 14.2 (StataCorp LP, College Station, TX, USA). A p value of <0.05 was considered statistically significant. Findings of this study were reported according to the SRTOBE guidelines (Strengthening the Reporting of Observational Studies in Epidemiology [17].

## Results

### Characteristics of the study sample

**Fig 1:**
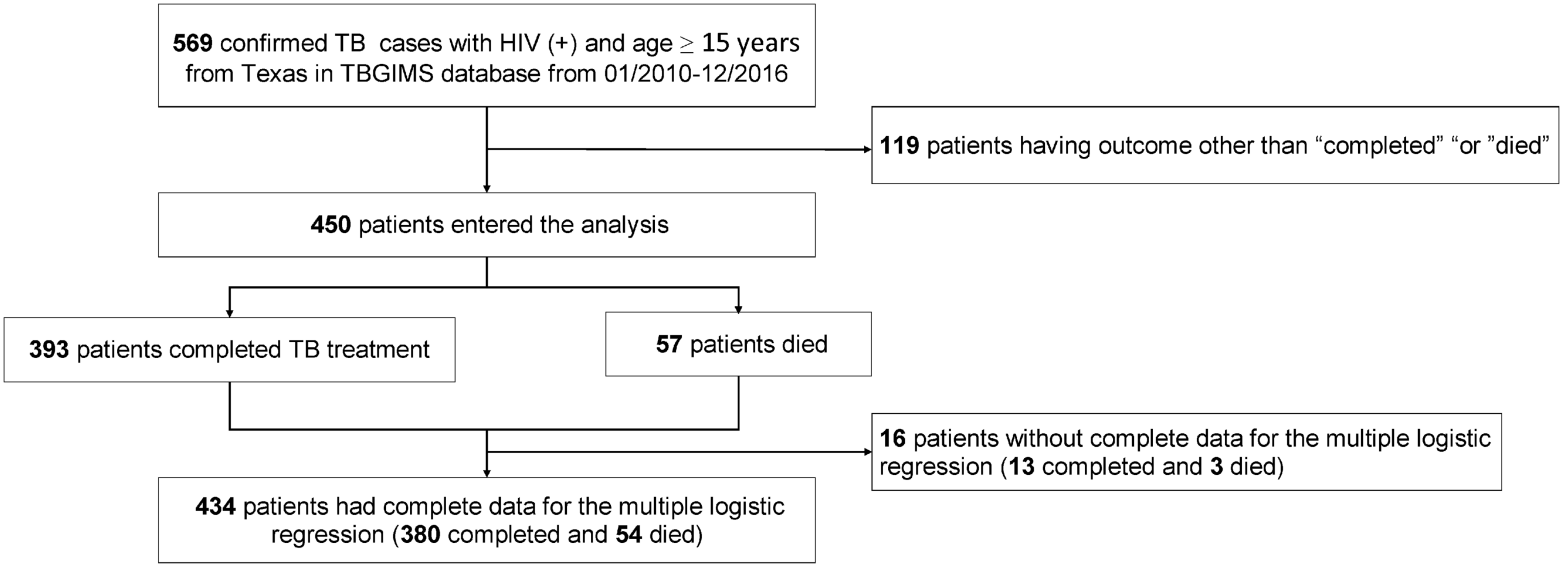
Flowchart of the study population. Footnote: TBGIMS, TB Genotyping Information Management System.

From January 2010 through December 2016, 569 HIV-infected adults in the state of Texas were confirmed with TB disease and reported in the National TB Surveillance System database. A total of 450 patients, including 57 individuals who died (12.7%) were used in the analysis after 119 patients with an outcome other than “completed” or “died” were excluded (Fig 1). There were no significant differences between characteristics of the excluded and included groups (Table 1). Data of the 434 included patients were used in the development and internal validation of the mortality prognostic scoring system.

**Table 1:** Demographic and clinical characteristics of the study population compared with those not included in the study.

	Included (N = 450)	Excluded (N = 119)	*P* Value*
Age (years)			0.91
15–44	256 (56.9%)	67 (56.3%)	
≥45	194 (43.1%)	52 (43.7%)	
Gender			0.15
Female	104 (23.1%)	20 (16.9%)	
Male	346 (76.9%)	98 (83.1%)	
Race/Ethnicity			0.24
White	44 (9.8%)	11 (9.2%)	
Black	216 (48.0%)	47 (39.5%)	
Hispanic	174 (38.7%)	54 (45.4%)	
Asian	14 (3.1%)	5 (4.2%)	
Other	2 (0.4%)	2 (1.7%)	
US-born			0.29
No	184 (40.9%)	55 (46.2%)	
Yes	266 (59.1%)	64 (53.8%)	
Resident of long-term care facility			0.99
No	443 (98.4%)	117 (98.3%)	
Yes	7 (1.6%)	2 (1.7%)	
Chronic kidney failure			0.25
No	443 (98.4%)	115 (96.6%)	
Yes	7 (1.6%)	4 (3.4%)	
Meningeal TB			0.30
No	424 (94.2%)	109 (91.6%)	
Yes	26 (5.8%)	10 (8.4%)	
Miliary TB			0.48
No	403 (91.0%)	107 (93.0%)	
Yes	40 (9.0%)	8 (7.0%)	
TB-CXR^†^			0.59
No	62 (14.3%)	17 (16.3%)	
Yes	372 (85.7%)	87 (83.7%)	
TB case verified by			0.93
Clinical case definition/provider diagnosis	81 (18.0%)	21 (17.6%)	
Positive culture, NAA^‡^ or smear	369 (82.0%)	98 (82.4%)	

Note: Values are in number and % unless otherwise specified.

*differences across groups were compared using the Chi-square or Fisher’s exact tests, as appropriate.

^†^TB-CXR: TB-specific abnormalities on chest radiograph.

^‡^NAA: Nucleic Acid Amplification.

### Development of the mortality prognostic score system

The crude associations between potential risk factors and mortality were examined using univariate logistic regression analyses (Table 2). The variable selection process using the Bayesian model averaging method suggested seven variables with prognostic significance for further investigation in the final multiple logistic regression model: age group, homelessness, resident of long-term care facility, meningeal TB, TB-CXR, TB diagnosis confirmed by positive culture or Nucleic Acid Amplification (NAA), *Mtb* culture without conversion or unknown conversion status during the TB treatment. Six variables were used in the development of the TB mortality risk score, all except for the homelessness variable, which was not significant in the final model. Weighted points were assigned to each of the final six risk factors using the linear transformation of the corresponding regression coefficient [(divided by the smallest β coefficient (1.07, age), multiplied by a constant (5), and rounded to the nearest integer, (Table 3)].

**Table 2:** Crude associations between potential risk factors and mortality.

	Total (N = 450)	Completed (*n* = 393)	Deceased (*n* = 57)	Unadjusted OR (95% CI)	*P* Value
Age ≥45 (years)	194 (43.1%)	157 (39.9%)	37 (64.9%)	2.78 (1.56, 4.97)	0.001
Male gender	346 (76.9%)	303 (77.1%)	43 (75.4%)	0.91 (0.48, 1.74)	0.78
Race/Ethnicity					
White	44 (9.8%)	37 (9.4%)	7 (12.3%)	1.22 (0.5, 2.99)	0.66
Black	216 (48.0%)	187 (47.6%)	29 (50.9%)	(ref)	
Hispanic	174 (38.7%)	155 (39.4%)	19 (33.3%)	0.79 (0.43, 1.46)	0.46
Asian	14 (3.1%)	12 (3.1%)	2 (3.5%)	1.07 (0.23, 5.05)	0.93
Other	2 (0.4%)	2 (0.5%)	0 (0.0%)	--	--
US-born	266 (59.1%)	227 (57.8%)	39 (68.4%)	1.58 (0.88, 2.87)	0.13
Homeless	61 (13.6%)	48 (12.2%)	13 (22.8%)	2.12 (1.07, 4.23)	0.03
Resident of correction institution	35 (8.3%)	33 (9.0%)	2 (3.7%)	0.39 (0.09, 1.67)	0.20
Resident of long-term care facility	7 (1.6%)	3 (0.8%)	4 (7.0%)	9.81 (2.14, 45.05)	0.003
IDU	38 (8.4%)	31 (7.9%)	7 (12.3%)	1.63 (0.68, 3.91)	0.27
Excess alcohol use within past 12 months	144 (32.0%)	126 (32.1%)	18 (31.6%)	0.98 (0.54, 1.78)	0.94
History of diabetes	21 (4.7%)	13 (3.3%)	8 (14.0%)	4.77 (1.88, 12.09)	0.001
Chronic kidney failure	7 (1.6%)	4 (1.0%)	3 (5.3%)	5.4 (1.18, 24.8)	0.03
TB site	395 (87.8%)	347 (88.3%)	48 (84.2%)	0.71 (0.33, 1.54)	0.38
Meningeal TB	26 (5.8%)	18 (4.6%)	8 (14.0%)	3.4 (1.4, 8.24)	0.01
Miliary TB	40 (9.0%)	32 (8.3%)	8 (14.3%)	1.85 (0.81, 4.25)	0.15
TB-CXR	372 (85.7%)	323 (85.0%)	49 (90.7%)	1.73 (0.66, 4.53)	0.27
Cavitation on CXR	63 (16.9%)	60 (18.6%)	3 (6.1%)	0.29 (0.09, 0.95)	0.04
Positive AFB smear	173 (41.4%)	153 (41.0%)	20 (44.4%)	1.15 (0.62, 2.15)	0.66
Positive MTB culture	280 (67.6%)	244 (66.3%)	36 (78.3%)	1.83 (0.88, 3.81)	0.11
Sputum culture not converted or unknown	211 (46.9%)	168 (42.7%)	43 (75.4%)	4.11 (2.18, 7.76)	<0.001
TB confirmed by positive culture or NAA	369 (82.0%)	317 (80.7%)	52 (91.2%)	2.49 (0.96, 6.46)	0.06
MDR-TB	1 (0.2%)	1 (0.3%)	0 (0.0%)	--	
East Asian lineage (L2)	75 (21.8%)	62 (20.9%)	13 (27.7%)	1.45 (0.72, 2.91)	0.30

IDU: injecting drug user; CXR: chest radiograph; NAA: Nucleic Acid Amplification; MDR-TB: Multi-drug resistant tuberculosis; excess alcohol use, having consumed five or more drinks on the same occasion on each of 5 or more days in the past 30 days, either self-reported or medically documented [12].

**Table 3:** Multiple logistic regression model and weighted point assignment.

Variable	β coefficient	Adjusted OR (95% CI)	*P* Value	Weighted Points
Age ≥45 (years)	1.07	2.91 (1.47, 5.78)	0.002	5
Resident of long-term care facility	2.54	12.69 (1.84, 87.44)	0.01	12
Meningeal TB	2.00	7.38 (2.42, 22.48)	<0.001	9
TB-CXR	1.38	3.97 (1.17, 13.5)	0.03	6
TB confirmed by positive culture or NAA	1.90	6.68 (2.33, 19.16)	<0.001	9
Culture not converted or unknown conversion status	2.19	8.9 (4.23, 18.74)	<0.001	10

Note: NAA: Nucleic Acid Amplification; Weighted points of a risk factor were calculated using a linear transformation of the corresponding β coefficient [was divided by the smallest β coefficient (1.07, age), multiplied by a constant (5), and rounded to the nearest integer]; Intercept = -6.994499.

$$\begin{array}{l} \text{Risk}\;\text{score}=5*\left[\text{Age}\geq 45\right]+12*\left[\text{Resident}\;\text{of}\;\text{long-term}\;\text{care}\;\text{facility}\right]+9*\left[\text{Meningeal}\;\text{TB}\right]\\ +6*\left[\text{TB-CXR}\right]+9*\left[\text{positive}\;\text{culture}\;\text{or}\;\text{NAA}\right]+10*\left[\text{Culture}\;\text{not}\;\text{converted}\;\text{or}\;\text{unknown}\right]. \end{array}$$

A prognostic score was calculated for individual patients based on the following formula:

$$\begin{array}{l} \text{Prognostic}\;\text{score}=5*\left[\text{Age}\geq 45\right]+12*\left[\text{Resident}\;\text{of}\;\text{long-term}\;\text{care}\;\text{facility}\right]+9*\left[\text{Meningeal}\;\text{TB}\right]\\ +\;6*\left[\text{TB-CXR}\right]+9*\left[\text{positive}\;\text{culture}\;\text{or}\;\text{NAA}\right]+10*\left[\text{Culture}\;\text{not}\;\text{converted}\;\text{or}\;\text{unknown}\right] \end{array}.$$

**Fig 2:**
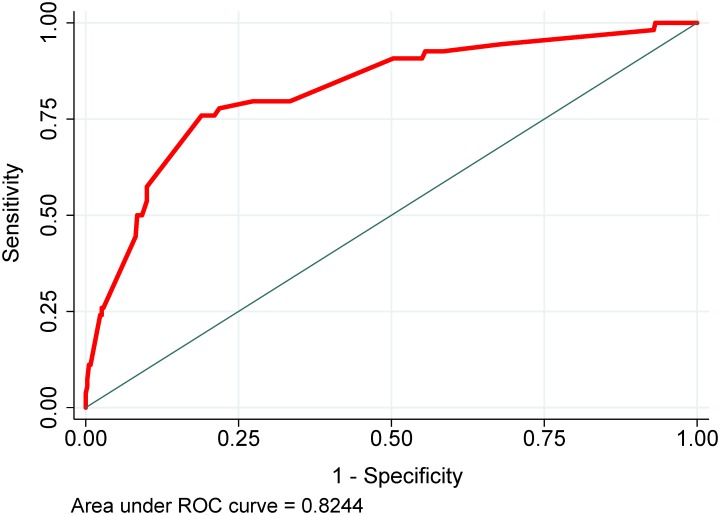
Receiver operating characteristic (ROC) curve.

All variables were binary with “No” = 0 and “Yes” = 1. Patients were divided into three groups that were significantly distinct in predictive risk for mortality: low-risk group (<20 points), medium-risk group (20–25 points), and high-risk group (>25 points). The mortality in low-, medium-, and high-risk groups were 2.6%, 11.9% and 44.4%, respectively (Table 4, Fig 2). The predicted probability of death during TB treatment can be calculated from the intercept (-6.994499) of the final model and corresponding regression coefficients of the variables included in the risk score based on the following formula:

$$\begin{array}{l} \text{Predicted}\;\text{probability}\;\text{of}\;\text{death}=-6.994499+1.069024*\left[\text{Age}\geq 45\right]\\ +2.541147*\left[\text{Resident}\;\text{of}\;\text{long-term}\;\text{care}\;\text{facility}\right]+1.998852*\left[\text{Meningeal}\;\text{TB}\right]\\ +1.37995*\left[\text{TB-CXR}\right]+1.899108*\left[\text{positive}\;\text{culture}\;\text{or}\;\text{NAA}\right]+2.186305*\left[\text{Culture}\;\text{not}\;\text{converted}\;\text{or}\;\text{unknown}\right]. \end{array}$$

**Table 4:** Mortality by risk group in patients having complete data for all variables of the multiple logistic regression model.

Risk group	*n* (%)	Mean score (±SD)	Mortality (%)	*P* Value *
Low-risk group (<20 points)	194 (43.1%)	15 (±2.2)	2.6%	<0.001
Medium-risk group (20–25 points)	177 (39.3%)	21.8 (±2.2)	11.9%	
High-risk group (>25 points)	63 (14.0%)	30.8 (±2.9)	44.4%	
Incomplete data for all variables of multiple logistic regression model	16 (3.6%)	--	18.8%	
All (N = 450)	450 (100%)	20.1 (±5.9)	12.7%	
*Discrimination assessment*				
AUC (95% CI), final model in development	0.82 (0.76, 0.89)			
AUC (95% CI), final model in bootstrap validation	0.80 (0.72, 0.88)			
AUC (95% CI), prognostic score alone	0.82 (0.76, 0.88)			
AUC (95% CI), prognostic score alone, bootstrap validation	0.79 (0.70, 0.87)			
*Calibration assessment*				
Hosmer-Lemeshow’s goodness of fit test, final model	Chi-square = 3.74; *P* Value = 0.71			
Hosmer-Lemeshow’s goodness of fit test, prognostic score alone	Chi-square = 4.25; *P* Value = 0.51			
*Overall performance assessment*				
Brier score, final model	0.09			
Brier score, prognostic score alone	0.09			

Comparisons of mortality between risk groups were conducted using Chi-square test.

*Overall p-value. A p<0.001 was also found for all pairwise comparisons among groups (i.e. low-risk vs. medium-risk, low-risk vs. high-risk and medium-risk vs. high-risk groups); a non-significant Hosmer-Lemeshow goodness of fit test indicates good calibration; Brier score: ranged 0–1, the smaller the score, the better performance.

### Performance and validation of the prognostic score

The final model had good discrimination in the development (AUC = 0.82; 95% CI 0.76, 0.89) and bootstrap validation (AUC = 0.80; 95% CI 0.72, 0.88) (Table 4, Fig 2). The ROC analysis using the prognostic score itself also provided good discrimination in both the development and bootstrap validation (AUC = 0.82; 95% CI 0.76, 0.89 and AUC = 0.79; 95% CI 0.70, 0.87, respectively). The prognostic model had good calibration with a non-significant Hosmer-Lemeshow chi-square of 3.74 (p = 0.71) and excellent overall performance with a Brier score of 0.09 (Table 4). Shrinkage statistic calculated using the repeated 10-fold cross-validation (250 replications) indicated an in-sample shrinkage of 1.4% (standard error 1.8). This result together with a non-significant Hosmer-Lemeshow goodness-of-fit test suggested the model fit well with the data. Compared with low-risk patients, patients in the medium- and high-risk groups had significantly higher odds of mortality during TB treatment (Table 5). Given that the multivariate analysis requires non-missing data for all the included variables for each patient, a sample of 434/450 (96.4%) patients having complete data for all six included variables were used in the final model and in the development of the scoring system. The comparison between 434 patients who were included in the final model versus 16 excluded patients due to incomplete data found no significant difference neither in the mortality (12.4% versus 18.8%, p = 0.46) nor in all demographic and clinical characteristics (S1 Table). Little’s chi-squared test for MCAR and CDM had non-significant p-values (0.13 and 0.44, respectively), which suggest that the missing values could be completely at random and do not influence on the outcome.

**Table 5:** Odds for death, by risk group (with bootstrap in estimating coefficient standard errors).

Risk group	OR (95% CI)	*P* Value
Low-risk group (<20 points)	(ref)	
Medium-risk group (20–25 points)	5.09 (1.88, 13.81)	0.001
High-risk group (>25 points)	30.24 (10.93, 83.66)	<0.001

### Online calculator application

We have created a free online application for our risk score calculator, which can be used on both android and iOS mobile devices. The calculator can be downloaded from the following link https://oaa.app.link/i0oYeyKsTK (registration for a free OpenAsApp account is required to access the calculator). The calculator provides a risk score (in points), risk group (low, medium or high), and probability of death (%) for an individual patient.

## Discussion

In this study, we developed and internally validated a simple prognostic scoring system to predict the mortality risk during TB treatment for TB/HIV co-infected patients in an area having low TB incidence (4.9/100,000) [4]. Our prognostic score was developed using population-based surveillance data in an exclusively HIV-infected population in a low TB-burden setting. Using only six variables, which are routinely collected in TB programs, our mortality predictive model achieves excellent discrimination and good calibration and therefore, can provide clinicians and public health professionals with more information regarding the patient’s risk of death during their treatment for TB disease. In order to enhance the practical implementation of the scoring system and to help allocate appropriate treatments and follow-up resources, we categorized patients into three distinctive risk groups. High-risk patients would need the most attention with urgent treatment and more aggressive medical support. Medium-risk patients could benefit from closer follow-up and prompt intervention if needed to prevent them from falling into aggravated health conditions. Low-risk group should be treated and managed as per routine protocols. Multiple approaches can be implemented to reduce a patient’s mortality risk. For example, early combination antiretroviral therapy (cART) could be considered for high-risk patients even though their CD4+ level ≥50 cells/mm3 as the cART has been shown to reduce up to 68% TB-related deaths in TB/HIV co-infected patients [18]. For individuals living in long-term care facilities, more aggressive nutritional support would be needed to improve the patient survival as these patients also often have other conditions that may increase the risk for TB mortality such as old age, poor living conditions, malnutrition and the presence of other comorbidities [19]. Educational sections for patients and their families toward managing the patient’s increased risk of mortality would need to be conducted to enhance the treatment adherence, improve the patient’s nutrition condition, and provide the knowledge of how to seek medical assistant when needed.

In our model, being a resident in a long-term care facility appeared to be the strongest predictor of mortality. Older age, poor nutrition condition, presence of other comorbidities and lack of family support could contribute to the morality risk for individuals living in long-term care facilities [20,21]. Delay in culture conversion has been suggested to be associated with a poor TB treatment outcome. Potential drug-resistant disease, failure to adhere to the treatment regimen and heavy initial bacillary load are among possible explanations for the adverse outcome [22, 23]. In our study, patients with TB meningitis had significantly higher odds of mortality, which is consistent with the observation of other authors [24]. Patients with TB-CXR (abnormal chest radiograph consistent with TB disease) and *Mtb* positive culture or NAA results had significantly higher mortality rates, nearly four times and seven times the odds for death compared with patients who had normal chest radiograph or negative cultures. It is possible that patients with TB-CXR and positive cultures may have a higher *Mtb* bacillary load and more disseminated lesions, which may increase the risk for death. A similar result has been described by Christensen et al. in their study in which pulmonary TB patients had an almost two-fold increased long-term mortality than extrapulmonary TB patients [25]. Although both TB-CXR and cavitation on CXR were evaluated in the initial multivariate model, only TB-CXR was significant. Additionally, TB/HIV co-infected patients may have a wide variety of radiographic findings rather than just cavitation [26]. Therefore, TB-CXR was included in the final model. The association between older age and worse outcome has also been observed by other authors [27,28]. Being significant in the univariate analysis, the diabetes and chronic kidney disease variables were evaluated in the initial multiple logistic regression model. However, these variables were not significant in multivariate analysis. Additionally, the model without diabetes nor chronic kidney disease had the same diagnostic performance as the model with these two variables included as confirmed by a non-significant Likelihood Ratio test result. Therefore, diabetes and chronic kidney disease were not included in our final model.

Our study has some limitations. First, the analysis excluded 119 (20.9%) out of 569 patients who had treatment outcome coded other than “completed” or “died”. While this exclusion may be prone to misclassification bias, the similarity in demographic and clinical characteristics between the excluded and included patients suggested that potential misclassification, if any, was minimal. Second, although antiretroviral therapy (ART) has been suggested as being strongly associated with the mortality reduction in TB/HIV co-infected individuals [7,29], ART use and some other important prognostic factors such as HIV viral load, CD4 cell count, time of death and cause of death are not available in the NTSS data, and thus prevented us from developing an even more robust model for this population. Assuming the majority of our TB/HIV co-infected patients received ART given the extensive HIV management programs in the U.S. and as our model was developed and validated in a low TB prevalence country, external validation of our prognostic score in different populations would be needed. Although patients in the high-risk group had more than 30 time the odds of death (OR 30.24, 95% CI 10.93, 83.66) compared with patients in the low-risk group, this finding should be interpreted cautiously given the wide confidence interval, which may be due to the small number of high-risk patients. Lastly, given that our study sample only had one patient with multi-drug resistant TB (MDR-TB), this variable was not examined in the multivariate analysis. Given MDR-TB is known high-risk factor for a high risk for death, external validation or updating of the model with the inclusion of MDR-TB as a variable using data from populations with a high proportion of MDR-TB should be conducted. Lastly, given the nature of surveillance data where certain self-reported information was originally obtained from interviewing TB patients, the possibility of recall bias cannot be completely ruled out.

In spite of the limitations, our study has many strengths such as the data were obtained from a state-wide population-based surveillance program over a 7-year time period, the variables used are routinely collected by any TB program, and the model has good discrimination and calibration in both development and validation. While the mortality in all TB disease patients (both positive and negative HIV status) in Texas was 5.0% (66/1334) in 2015 [4], the significantly higher mortality rate in HIV-positive TB patients found in this study (12.7%, 57/540) also raises questions regarding the need for having better management strategies for this high-risk group of patients. In addition, with the availability of a free and convenient calculator app, which can be accessed from any android and iOS devices, clinicians and health professionals can easily use our scoring system in their daily practice in order to facilitate their decision-making process.

## Conclusion

The present study developed and internally validated a simple and practical prognostic scoring system using the population-based surveillance data to predict mortality during TB treatment in TB/HIV co-infected patients. This TB mortality scoring system could help identify TB/HIV co-infected patients who have an increased risk of mortality. External validation of our risk score system using the provided formula in similar settings of low TB/HIV burden would be necessary.

## Supporting information

S1 Table Demographics, clinical characteristics and outcome between patients included (n = 434) versus excluded from the multiple logistic regression model (n = 16). Note: Values are in number and % unless otherwise specified; *differences across groups were compared using the Chi-square or Fisher’s exact tests as appropriate. TB-CXR: TB-specific abnormalities on chest radiograph. NAA, Nucleic Acid Amplification.
(PDF)
