# Supplementary material for: Prognostic score to predict mortality during TB treatment in TB/HIV co-infected patients
Source: PLoS One. 2018 Apr 16;13(4):e0196022. doi: 10.1371/journal.pone.0196022 (PMC5901929; doi:10.1371/journal.pone.0196022)
Supplement: S1 Table — Note: Values are in number and % unless otherwise specified; *differences across groups were compared using the Chi-square or Fisher’s exact tests as appropriate. TB-CXR: TB-specific abnormalities on chest radiograph. NAA, Nucleic Acid Amplification. (PDF) [file pone.0196022.s001.pdf]

S1 Table. Demographics, clinical characteristics and outcome between patients included (n=434) versus excluded from the multiple logistic regression model (n=16)

	Total (N = 450)	Excluded (*n*= 16)	Included (*n*= 434)	p-value*
**Age (years)**				0.14
15-44	256 (56.9%)	12 (75.0%)	244 (56.2%)	
≥45	194 (43.1%)	4 (25.0%)	190 (43.8%)	
**Gender**				0.16
Female	104 (23.1%)	6 (37.5%)	98 (22.6%)	
Male	346 (76.9%)	10 (62.5%)	336 (77.4%)	
**Race/Ethnicity**				0.62
White	44 (9.8%)	0 (0.0%)	44 (10.1%)	
Black	216 (48.0%)	8 (50.0%)	208 (47.9%)	
Hispanic	174 (38.7%)	8 (50.0%)	166 (38.2%)	
Asian	14 (3.1%)	0 (0.0%)	14 (3.2%)	
Other	2 (0.4%)	0 (0.0%)	2 (0.5%)	
**US-born**				0.81
No	184 (40.9%)	7 (43.8%)	177 (40.8%)	
Yes	266 (59.1%)	9 (56.3%)	257 (59.2%)	
**Resident of long-term care facility**				1.00
No	443 (98.4%)	16 (100.0%)	427 (98.4%)	
Yes	7 (1.6%)	0 (0.0%)	7 (1.6%)	
**Chronic kidney failure**				1.00
No	443 (98.4%)	16 (100.0%)	427 (98.4%)	
Yes	7 (1.6%)	0 (0.0%)	7 (1.6%)	
**Meningeal TB**				0.31
No	424 (94.2%)	16 (100.0%)	408 (94.0%)	
Yes	26 (5.8%)	0 (0.0%)	26 (6.0%)	
**TB-CXR**				
No	62 (14.3%)	0 (0.0%)	62 (14.3%)	NA
Yes	372 (85.7%)	0 (0.0%)	372 (85.7%)	
**TB case verified by**				0.21
Clinical case definition/provider diagnosis	81 (18.0%)	1 (6.3%)	80 (18.4%)	
Positive culture, NAA or smear	369 (82.0%)	15 (93.8%)	354 (81.6%)	
**Patient outcome**				0.46
Treatment completed	393 (87.3%)	13 (81.3%)	380 (87.6%)	
Dead	57 (12.7%)	3 (18.8%)	54 (12.4%)	

Note: Values are in number and % unless otherwise specified; *differences across groups were compared using the Chi-square or Fisher's exact tests as appropriate. TB-CXR: TB-specific abnormalities on chest radiograph. NAA, Nucleic Acid Amplification.
